# MicroRNA -148 alleviates cardiac dysfunction, immune disorders and myocardial apoptosis in myocardial ischemia-reperfusion (MI/R) injury by targeting pyruvate dehydrogenase kinase (PDK4): Myocardial protection of miR-148/PDK4 in immature rats.

**DOI:** 10.1080/21655979.2021.1965812

**Published:** 2021-09-14

**Authors:** Qi Yin, Ping Wang, Xiaohua Wu

**Affiliations:** Department of Health care center, Hainan People’s Hospital, Haikou, Hainan, China

**Keywords:** Mir-148, pdk4, cardiac dysfunction, immune disorder, myocardial injury

## Abstract

Ischemic heart disease in children may be induced by varied factors, and there is no corresponding systematic treatment up to now. This study aims to investigate the effects of microRNA (miR)-148 on myocardial injury in immature rats with myocardial ischemia-reperfusion (MI/R) injury. In this study, MI/R model was established by ligating the coronary artery of heart. The results showed that miR-148 alleviated myocardial injury and rescued relevant parameters (mean ventricular systolic blood pressure (MAP), left ventricular systolic blood pressure (LVSP), heart rate (HR), creatine kinase-MB (CK-MB), cTn1 and Mb in immature rats with MI/R injury. Besides, miR-148 improved the immune dysfunction induced by MI/R through increasing the number of interleukin (IL)-10^+^ cells and reducing the number of inducible nitric oxide synthase (iNOS)^+^ cells. In addition, miR-148 relieved the apoptosis of cardiomyocytes induced by MI/R through inhibiting the expression of Bax and elevating the expression of Bcl-2. Further molecular mechanism indicated that pyruvate dehydrogenase kinase 4 (PDK4) was the downstream target of miR-148, which was further confirmed by dual luciferase reporter assay and related expression detection. Accordingly, silenced PDK4 attenuated cardiac dysfunction, immune disorder and myocardial apoptosis in immature rats and enhanced the ability of antioxidant enzymes. What is more, activated SMAD pathway induced by MI/R injury was then blocked by silenced PDK4. Taken together, our study demonstrated that overexpressed miR-148 relieved cardiac dysfunction, immune disorder and cardiomyocyte apoptosis in immature MI/R rats by PDK4 inhibition, which provided novel targets for MI/R injury treatment.

## INTRODUCTION

Children with congenital heart disease are less athletic and are always accompanied by behavioral, emotional and cognitive disorders in childhood [1], adolescence and even in adulthood [2]. Besides, children with congenital heart disease are more likely to develop into mental disorders and have poor quality of life [3]. Myocardial ischemia is a multi-factor pathophysiological state involving complex and specific interactions between coronary arteries and myocardium [4]. Decreased level of high-energy phosphate decreases and acidosis are common in myocardial ischemia, which further lead to weakened myocardial contractility and cardiac function. The protection of myocardium is closely related to the metabolism, structure and function of cardiomyocytes [5]. However, the protective effect of mature myocardium in infants is significantly different from that of immature myocardium. Therefore, it is of great significance to explore the protective mechanism of immature myocardial ischemia-reperfusion (MI/R) in children with congenital heart disease.

MicroRNAs (MiRNAs) are cell-specific non-coding RNAs which regulate gene expression at the post-transcriptional level [6]. In zebrafish, the miR-133 family and miR-101A regulate cardiac regeneration by targeting cell cycle factors [7]. Comparative transcriptome maps of damaged zebrafish and mouse hearts further confirmed that miR-26a was a negative regulator of cardiomyocyte proliferation and it had great potential in targeted therapy after myocardial infarction [8]. Up-regulated MiR-148a has a positive regulatory effect on the myocardial differentiation of human bone marrow mesenchymal stem cell by targeting methyltransferase 1 gene [9]. Besides, miR-148a is a new kind of myogenic miRNA related to myogenic differentiation and the expression of miR-148a is up-regulated during the differentiation of C2C12 myoblasts [10]. In zebrafish heart, miR-148 has been identified as the highest regulator of cardiac regeneration [11]. Inflammatory response plays a very important role in MI/R injury. The level of inflammatory cell activation is directly related to the degree of MI/R injury and the number of myocardial cell necrosis [12]. Previous study pointed out that high level of miR-148a in macrophages regulated the host immune response [13]. Thus, miR-148 is closely associated with cardiomyocyte differentiation.

Pyruvate dehydrogenase kinase 4 (PDK4) is a key enzyme regulating the activity of pyruvate dehydrogenase complex and a key regulator of pyruvate oxidation and glucose homeostasis [14]. Previous study found that PDK4 inhibition increased myocardial glucose uptake after myocardial I/R, thus effectively promoting cardiac function recovery [15]. Here, the protective effects of PDK4 in MI/R injury and the targeting relationship between miR-148 and PDK4 were explored.

Up to now, the effects and potential molecular mechanism of miR-148 in myocardial protection in children with congenital heart disease are still unclear. Besides, previous study has never explored the association between immune system and miRNA in MI/R injury. This study investigated the effects of miR-148 on cardiac dysfunction, immune disorder and apoptosis in immature rats with MI/R injury. We aim to more clearly reveal the effects of miRNA in MI/R injury, and provide effective target for MI/R injury treatment.

## MATERIALS AND METHODS

### Cell transfection

MiR-148 Agomir and Agomir-negative control (NC), Ad-PDK4 and Ad-NC were purchased from Guangzhou RiboBio Technology Co., LTD. In vitro experiments, HEK-293 T cells served as the control group (CONTROL). Transfection of miRNA agomirs: Strictly following the manufacturer’s product instructions, Mir-148 agomir and agomir-NC were transfected into HEK-293 T cell lines through Lipofectamine®2000 (Invitrogen, USA). The transfection efficiency was detected by RT-qPCR for 48 h after transfection. Recombinant adenovirus transfection: miR-148 agomir and AD-PDK4 were respectively or jointly fully mixed with the transfection reagent, and incubated with the cells for 8 h after 25 min. After transfection for 48 h, HEK-293 T cells were collected for further analysis.

### Animals

48 healthy 2-week-old Sprague-Dawley (SD) rats (weighing 60 ~ 75 g) were purchased from the Experimental Animal Center of Guangzhou University with the production license Number SCXK (Yue) 2018–0002. According to local government regulations, the experiment was approved by the Animal Ethics Committee. The rats were fed at a temperature of 18 ~ 25 °C and a humidity of 50%-70%, and the model was made after 7 days of adaptive feeding.

### Grouping and MI/R model in SD rats

In this study, in order to investigate the effects of miR-148 on MI/R immature rats, the immature rats were divided into the following four group *in vivo* expreriments: Sham+agomir-NC, Sham+miR-148 agomir, MI/R+ agomir-NC and MI/R+ miR-148 agomir. To further study the effects of miR-148 and PDK4 on MI/R immature rats, four groups were divided as following: MI/R, MI/R+ miR-148 agomir, MI/R+ Ad-PDK4 and MI/R+ PDK4+ miR-148. The MI/R injury model was established as previously described [16].

SD immature rats were injected intravenously with ketamine (80 mg/kg) and Xylazine (20 mg/kg). Heparin (1500 IU/kg) was also administered intravenously to prevent thrombosis in the coronary arteries. Make an incision in the left chest to separate the muscles for exposing the ribs. A small incision was made in the intercostal space of the fourth and fifth ribs. Then the chest was opened to reveal the heart, and the left anterior descending branch of the left coronary artery was ligated for 30 min through suture, and the coronary blood flow was restored for 120 min. Thus, the model of ischemia-reperfusion injury was formed. The criteria for successful MI/R model construction refer to the previous literature [17]. Only after thoracotomy was performed in Sham group, ligation lines were crossed under the anterior descending branch of the left ventricle of coronary artery and ligation was performed. MiR-148 agomir or/and Ad-PDK4 were injected with 50 μL miR-148 agomir or/and Ad-PDK4 (viral titer 1.2 × 10^10^ TU/mL) at 4 points above, below, left and right of the left ventricular free wall of rats in MI/R+ miR-148 agomir, MI/R+ Ad-PDK4, MI/R+ PDK4+ miR-148 groups. Sham+agomir-NC and Sham+miR-148 agomir groups were injected with 50 μl blank controls in the same way. Close the chest incision and close the skin incision.

### The measurement of MAP, LVSP and HR

The hemodynamic parameters mean arterial pressure (MAP), left ventricular systolic pressure (LVSP) and heart rate (HR) were observed and recorded continuously during the whole I/R using the PowerLab biological signal acquisition and analysis system (ADI Company, Australia). The data were continuously measured for 5 times (5 min before ischemia, 0 min and 30 min after ischemia, and 30 min and 120 min after reperfusion) and the mean value was calculated.

### Detection of MDA, GSH and SOD levels

The activities of oxidative stress related enzymes malonyldialdehyde (MDA), glutathione (GSH) and superoxide dismutase (SOD) were evaluated by corresponding detection kits according to the instructions. The detection kits of MDA, GSH and SOD were purchased from Nanjing Jiancheng Bioengineering Institute.

### The measurement of CK-MB, cTnΙ, Mb, iNOS and IL-10 by ELISA

After the hemodynamic test, blood was collected from the abdominal aorta and naturally coagulation was performed for 30 min at 4 °C, 4500 r /min× 10 min at 4°C. The serum was divided and stored at −70°C. The content of creatine kinase isoenzyme (CK-MB), Troponin I (cTnΙ), myoglobin (Mb), inducible nitric oxide synthase (iNOS) and interleukin (IL)-10 was detected by ELISA kits (R&D Systems USA), respectively.

### Hematoxylin-Eosin (HE) staining

After all the blood related experiments were completed, the rats were killed through cervical dislocation under anesthesia (intraperitoneal injection of sodium pentobarbital at the dose of 30 mg/ kg). The HE staining was conducted according to standard protocols as previously described [18]. Corresponding tissue were collected for further experiments. Xylene dewaxed tissue sections were performed twice for 10 min each. Soak in 100%, 90%, 80% and 70% ethanol for 3 min successively. Then, hematoxylin staining was used for 2 min, 1% hydrochloric acid ethanol was differentiated for 10 sec, diluted ammonia reflux for 20 sec, and eosin staining was used for 3 min. After each of the above steps, rinse with deionized water for 3 times, 1 min each time. Then, the water was dehydrated by 70%, 80%, 90% and 100% ethanol in turn, with a gradient of 5 min each. Xylene was transparent for 2 times, 10 min each time, and then sealed with neutral resin. E100 microscope (Nikon, Japan) observed staining results and took photos for recording.

### Masson staining

The masson staining was conducted as previously described [19]. The cardiac tissues were firstly fixed with 10% formaldehyde for 24 hours at room temperature. After decalcified, dehydrated, permeabilized with xylene, the tissues were embedded in wax and finally cut into 5‐μm‐thick sections. The cell nucleus was dyed for 5 minutes using the Wiegert’s iron hematoxylin solution (Sigma Aldrich, St. Louis, MO, USA). After rinsed with distilled water for at least 3 times, the sections were stained with 0.7% Masson-Ponceau-acid fuchsin staining solution (Sigma-Aldrich) for 10 minutes at room temperature. Then, the sections were rinsed in 2% glacial acetic acid and differentiated in phosphomolybdic acid for 4 minutes. The sections were directly stained with 2% aniline blue dye solution (Sigma-Aldrich). Following dehydrating with ethanol series, clearing with xylene and mounting with neutral resins, images of the stained sections were captured with a light microscope.

### CD4^+^iNOS^+^ and CD4^+^IL-10^+^ cells in peripheral blood were sorted by flow cytometry

Total of 1 × 10^6^ cells were added to PBS. 100 L 1% fetal bovine serum and 1 g CD16/CD32 were placed on ice and incubated for 30 min to block the nonspecific Fc interaction, then PC-CD4 and APC-iNOS or FITC-IL-10 antibodies were used to tag the cells for 1 h. The labeled cells were resuspended in PBS in 1% FBS and analyzed by flow cytometry (BD, FACSAria II). Isotype IgG and unstained cells were used as negative controls. Using the same setting, CD4 and iNOS or IL-10 labeled cells were sorted by flow cytometry to obtain CD4^+^iNOS^+^ and CD4^+^IL-10^+^ subsets. PI (1 g/mL) was added to the cell suspension to remove the separated dead cells.

### TUNEL assay

TUNEL assay (C1098, Beyotime, Jiangsu, China) was used to detect the apoptosis of tumor tissues according to the manufacturer’s instruction. The stained tissue was observed under a E100 microscope.

### RNA pull down assay

The RNA pull down assay was conducted as previously described [20]. Biotin was attached to the 3’-end of miR-148 or the control. 1 × 10^6^ HEK-293 T cells were transfected with 100 pmol Bio-miR-148, or negative control using Lipofectamine 2000 according to the instruction. After 48 hours, the cells were centrifuged at the speed of 1000 rpm. After washed three times using PBS, the cell pellets were re-suspended using 0.7 ml lysis buffer (5 mM MgCl2, 100 mM KCl, 20 mM Tris (pH7.5), 0.3% NP-40, 50 U of RNase OUT (Invitrogen, USA)), complete protease inhibitor cocktail (Roche Applied Science, IN) on ice for 10 min. Then, the cell lysate was isolated through centrifugating at 10,000 g for 10 min. The level of PDK4 in the pull down of Bio-miR-148 or the control was detected through real-time PCR.

### RT-qPCR

Total RNA was extracted from rat myocardial tissue using Animal Tissue Total RNA Extraction Kit (Tiangen, Beijing, China). RT-qPCR was performed using Quant One Step RT-qPCR Kit (SYBR Green) (Tiangen, Beijing, China). SYBR Green Master Mix (Thermo Fisher) is used in ABI Prism 7000 Systems (Applied Biosystems, USA). The thermocycling conditions were as follows: A holding step at 95°C for 30 sec, and 40 cycles at 95°C for 5 sec and 60°C for 30 sec. The primers used for miRNA detection by RT-qPCR were designed based on sequences provided by the Sanger Center miRNA Registry. The U6 was used as an endogenous control for miR-148 expression levels. All primers of mRNA were designed as follows: PDK4 forward 5’-AACACCAGGAAAATCAGCC-3’, reverse 5’-AAAACCAGCCAAAGGAGC-3’; GAPDH forward 5’-ACCCAGAAGACTGTGGATGG-3’, reverse 5’-TCTAGACGGCAGGTCAGGTC-3’. The result was normalized to GAPDH and calculated with the 2^−ΔΔCt^ [21].

### Western Blot

The proteins in cells or tumors were isolated by 10% sodium dodecyl sulfate polyacrylamide gel (Solarbio, Beijing, China) and transferred to polyvinylidene fluoride membrane (Solarbio, Beijing, China). After being blocked by 5% skim milk, the protein was incubated with the following first antibodies: anti-iNOS, ab178945, 1:1000; IL-10, ab133575, 1:1000; PDK4, ab110336, 1:1000; GAPDH, ab8245, 1:2000 (Cell Signaling Technology, USA) at 4 °C overnight. The sheep anti-rabbit IgG-HRP (Solarbio, Beijing, China) was washed with PBS and incubated for 1 h. The strips were observed with the ECL chemiluminescence detection kit (Solarbio, Beijing, China) [22].

### Cell transfection

The siRNA PDK4 (50pM), siRNA NC (50pM), miR-148 inhibitor (100 nM), NC inhibitor (100 nM) were obtained from RiboBio. NC mimic (50 nM) and miR-148 mimic (50 nM) were purchased from Shanghai IBSBIO Co., Ltd. The transfection was conducted using Lipofectamine 2000 (Life Technologies, Gaithersburg, MD, USA) according to the manufacturer’s instructions.

### Dual luciferase reporter assay

The target between PDK4 and miR-148 was predicted using Targetscan (http://www.targetscan.org/vert_71/). The 3’UTR fragments of PDK4 (WT and MUT) were constructed into luciferase reporting vector (pmirGLO) (Promega, USA): Luc-PDK4 3’UTR WT (PDK4-WT), Luc-PDK4 3’UTR MUT (PDK4-MUT). Then, HEK-293 T cells were co-transfected with agomir-NC or miR-148 agomir/inhibitor-NC or miR-148 inhibitor and pmirGLO (WT and MUT) [23]. Luciferase activity was determined by a dual-luciferase reporter assay kit (Promega, USA).

### Statistical analysis

Experiments were repeated at least three times and the data are presented as the means ± standard deviation. Statistical comparisons between two groups were conducted using t test. Statistical comparisons between different groups were conducted using a two-way analysis of variance followed by Bonferroni post hoc test using SPSS version18.0. P < 0.05 was considered to indicate a statistically significant difference.

## RESULTS

### Overexpression of miR-148 alleviates cardiac dysfunction induced by MI/R in immature rats

**Figure 1.**
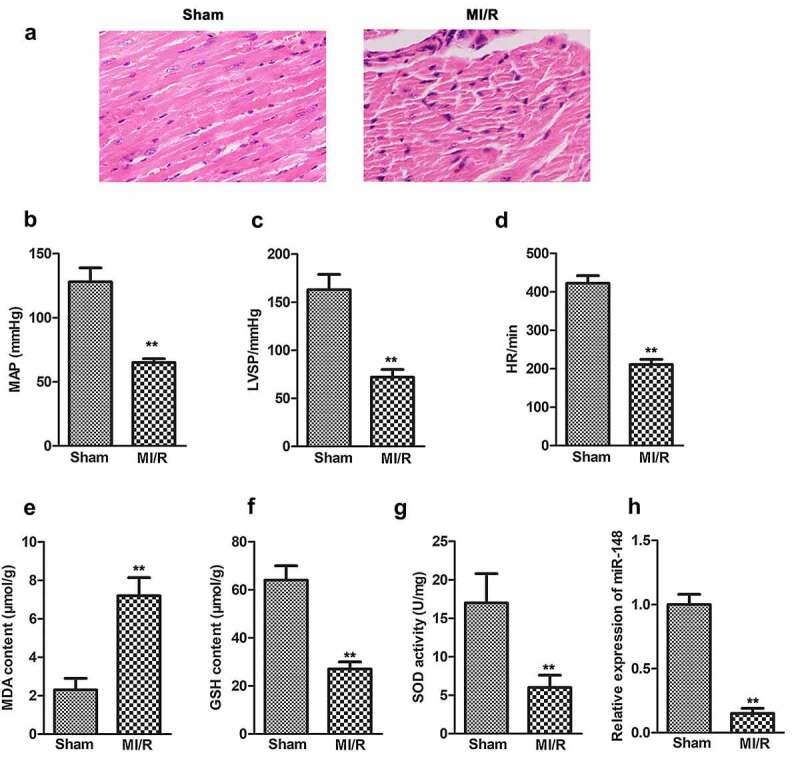
**Comparison of cardiac dysfunction related index in MI/R group and sham group**. (a) Pathological injury was measured by HE staining (×200). (b) MAP value. (c) LVSP value. (d) HR value. (e) The content of MDA. (f) The content of GSH. (g) The content of SOD. (h) Relative expression of miR-148 in MI/R group and sham group was measured by qPCR. The data is shown as means ± SD, **P < 0.01 vs. Sham group

**Figure 2.**
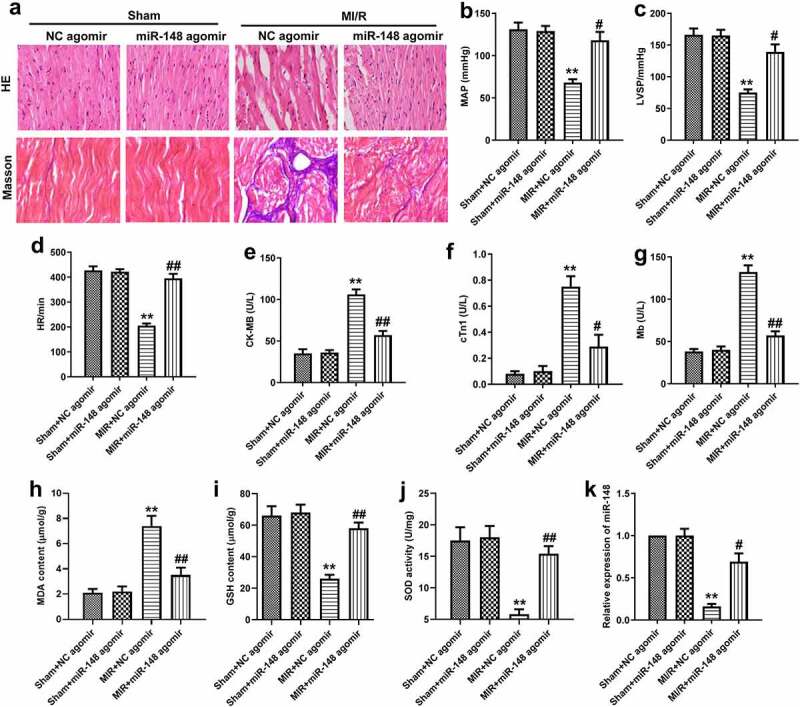
**Overexpression of miR-148 alleviates cardiac dysfunction induced by MI/R in immature rats**. (a) Relative expression of miR-148 was measured by qPCR. (b) Pathological injury was measured by HE staining (×200) and the Masson staining (×200). (c) MAP value. (d) LVSP value. (e) HR value. (f) The content of CK-MB. (g) The content of cTn1. (h) The content of Mb. (i) The content of MDA. (j) The content of GSH. (k) The content of SOD. The data is shown as means ± SD, **P < 0.01 vs. Sham + agomir-NC group, ^#^*P* < 0.05 vs. MI/R + agomir-NC group, and ns means no significant difference

In order to investigate the effect of miR-148 on the cardiac function of immature rats with MI/R injury, agomir-NC or miR-148 agomir were transferred into rats in sham group and MI/R group respectively. Myocardial injury was observed by HE staining. As shown in Figure 1a, the edge of cardiomyocytes in sham group was relatively intact. The myocardial fibers were arranged neatly. No inflammatory cell infiltration was found in myocardial interstitium. The cardiomyocytes of rats in MI/R group were swollen and deformed. The nucleus was concentrated or dissolved. Most of the myocardial fibers were necrotic and the boundary was unclear. Some myocardial fibers are twisted and broken. When miR-148 was overexpressed, cardiomyocyte edema was significantly alleviated (Figure 2a). Inflammatory cell infiltration also decreased significantly. Myocardial fiber necrosis was significantly alleviated (Figure 2a). The myocardial fibers were arranged neatly. Cardiac function indexes such as left ventricular systolic blood pressure (LVSP), mean arterial pressure (MAP) and heart rate (HR) were measured. The values of LVSP, MAP and HR decreased significantly in MI/R group (Figure 1b, 1c and 1d). At the same time, obviously elevated expression level of antioxidant index MDA and suppressed expression level of GSH and SOD were also found in MI/R group compared with the control, exhibiting that the antioxidant function of myocardial cells is largely decreased (Figure 1e, 1f and 1g). In addition, expression of miR-148 was suppressed in the MI/R group and suppressed expression of miR-148 was rescued by the addition of miR-148 agomir (Figs. 1H and 2a). However, the results of HE and Masson staining showed that the damaged cardiomyocytes and inflammation were effectively relieved by overexpressed miR-148 (Figure 2b). Up-regulation of miR-148 could reverse the decreased level of MAP, LVSP, and HR induced by MI/R (Figure 2c, 2d and 2e). The indexes of myocardial injury, such as creatine kinase isoenzyme (CK-MB), troponin (CTn) and myoglobin (Mb) were also measured. The values of CK-MB, cTn1 and MB were significantly increased in MI/R group, but overexpression of miR-148 reversed the increase of CK-MB, cTn1 and Mb induced by MI/R (figure 2f, 2g and 2h). At the same time, decreased antioxidant function of myocardial cells is largely resumed by overexpressed miR-148 through down-regulating the level of MDA and elevating the level of GSH and SOD (Figure 2i, 2j and 2k).

### Overexpression of miR-148 alleviates immune disorder caused by MI/R

**Figure 3.**
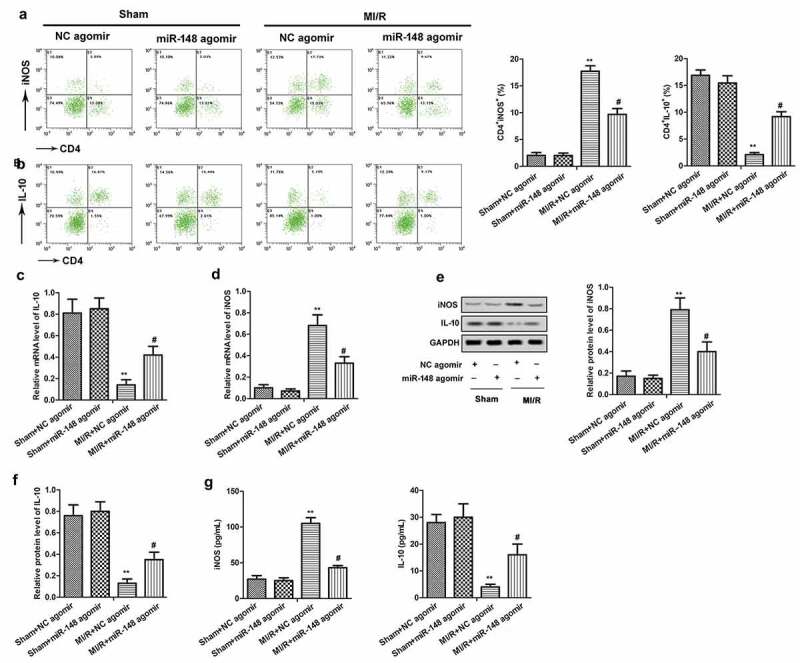
**Overexpression of miR-148 alleviated immune disorder caused by MI/R**. (a-b) CD4^+^iNOS^+^ and CD4^+^IL-10^+^ cells in peripheral blood were sorted by flow cytometry. (c) Relative expression of IL-10 was measured by qPCR. (d) Relative expression of iNOS was measured by qPCR. (e-f) Relative protein levels of iNOS and IL-10 were measured by Western Blot. (g) The level of iNOS was measured by ELISA. (h) The level of IL-10 was measured by ELISA. The data is shown as means ± SD, **P < 0.01 vs. Sham + agomir-NC group, ^#^*P* < 0.05 vs. MI/R + agomir-NC group, and ns means no significant difference

To investigate the immunomodulatory effect of miR-148 on the peripheral blood of immature rats with MI/R injury, the inducible nitric oxide synthase (iNOS) and interleukin-10 (IL-10) positive cells in the immune system were detected by flow cytometry. As shown in Figure 3a-B, the number of CD4^+^iNOS^+^ cells increased significantly, while the number of CD4^+^IL-10^+^ cells decreased significantly in MI/R group compared with the sham group. Then, up-regulation of miR-148 reversed these above results. The levels of iNOS and IL-10 in peripheral blood of rats were also detected by RT-qPCR and ELISA. As shown in Figure 3c, 3d, and 3g, the expression level of iNOS in peripheral blood of immature rats was increased, while the level of IL-10 was decreased in MI/R injury group compared with the control. However, the change in the expression of iNOS and IL-10 was reversed by overexpressed miR-148. After the addition of miR-148 agomir, the level of iNOS was decreased, while the level of IL-10 was increased. The expression of iNOS and IL-10 in myocardium was also detected by western blot. The results showed that the expression of iNOS was up-regulated and the expression of IL-10 was down-regulated in immature rats with MI/R injury, and the overexpression of miR-148 reversed these results (Figure 3e, 3f). These above results suggest that the overexpression of miR-148 alleviated the immune disorder of immature rats with MI/R injury.

### Overexpression of miR-148 alleviates cardiomyocyte apoptosis induced by MI/R

**Figure 4.**
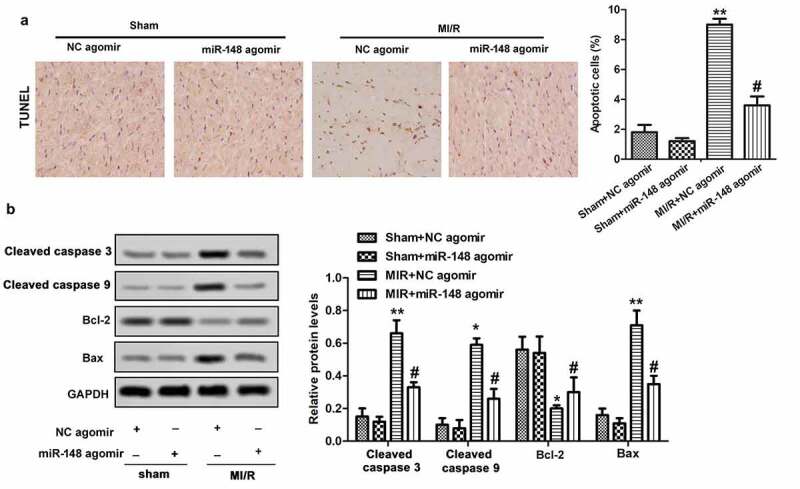
**Overexpression of miR-148 alleviates cardiomyocyte apoptosis induced by MI/R** (a) Cell apoptosis was measured by TUNEL. (b) Relative protein levels of Bcl-2 and Bax were measured by Western Blot. The data is shown as means ± SD, **P < 0.01 vs. Sham + agomir-NC group, ^#^*P* < 0.05 vs. MI/R + agomir-NC group, and ns means no significant difference

Apoptosis is an important phenotype of myocardial injury, thus we investigated the effects of overexpressed miR-148 on cardiomyocyte apoptosis. In this study, TUNEL method was used to detect the effects of miR-148 on cardiomyocyte apoptosis induced by MI/R in immature rats. The results showed obviously increased apoptosis rate of cardiomyocytes in immature rats with MI/R injury. However, the apoptosis rate decreased rapidly with forced overexpression of miR-148 (Figure 4a). As we all know, Bcl-2 and Bax are important protein related to apoptosis inhibition and promotion, respectively [24]. Western Blot showed that MI/R injury led to suppressed Bcl-2 expression and elevated Bax expression. However, overexpression of miR-148 suppressed apoptosis by increasing the expression of Bcl-2 and suppressing the expression of Bax (Figure 4b). Taken together, these results suggest that overexpression of miR-148 inhibited cardiomyocyte apoptosis in immature rats with MI/R injury.

### MiR-148 targets PDK4 gene

**Figure 5.**
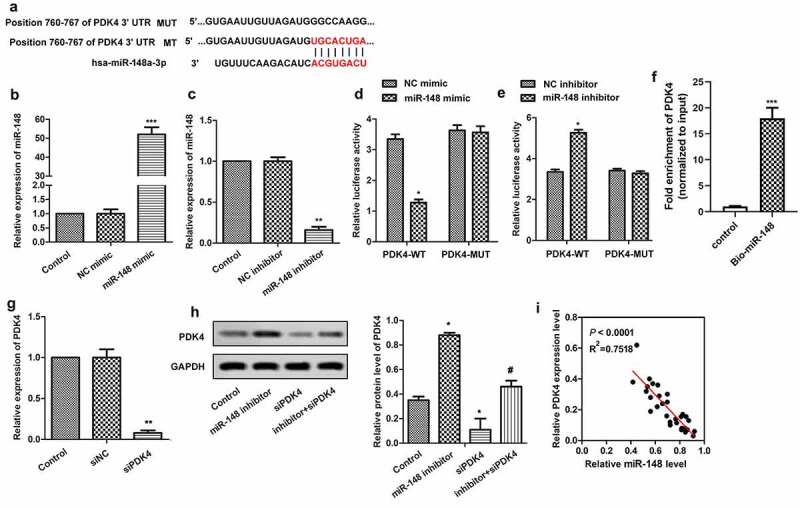
**MiR-148 targets PDK4 gene**. (a) Potential target sites for miR-148 and PDK4. (b) Relative expression of miR-148 in HEK-293 T cells transfected with miR-148 agomir or agomir-NC. **P < 0.01 vs. Control group (c) Relative expression of miR-148 in HEK-293 T cells transfected with miR-148 inhibitor or Inhibitor-NC. **P < 0.01 vs. Control group (d-e) Relative luciferase activity in HEK-293 T cells was measured by dual luciferase reporter assay. *P < 0.05, **P < 0.01 vs. PDK-WT + agomir-NC group or DK-WT + inhibitor-NC group (f) The targeting relations of PDK4 and miR‐148 were confirmed by RNA pull‐down assay. Endogenous PDK4 was enriched specifically in miR‐148 probe detection compared with control group. (g)Relative expression of PDK4 in HEK-293 T cells transfected with siPDK4 or siNC was measured by RT-qPCR. **P < 0.01 vs. Control group (h) Relative protein level of PDK4 in HEK-293 T cells transfected with si-PDK4 or/and miR-148 inhibitor was measured by Western Blot. (i) Correlation analysis about the expressions of PDK4 and miR-148. The data is shown as means ± SD, *P < 0.05, **P < 0.01 vs. Control group, ^#^*P* < 0.05 vs. Ad-PDK4 group, and ns means no significant difference

The target gene of MiR-148 was further explored. The potential-binding sites of miR-148 and PDK4 were predicted by bioinformatics software, which revealed that PDK4 might be the target gene of miR-148 (Figure 5a). In order to verify the conjecture, miR-148 was overexpressed and silenced by transfecting miR-148 agomir and miR-148 inhibitor into cells, respectively. As shown in Figure 5b and 5C, the expression of miR-148 was effectively up-regulated or down-regulated with the transfection of miR-148 agomir (Figure 5b) or miR-148 inhibitor (Figure 5c) compared with the control group. Besides, Dual luciferase reporter assay further confirmed the relationship between miR-148 and PDK4. The luciferase activity was decreased significantly after the co-transfection of PDK4-WT (wild type) and miR-148 agomir (Figure 5d), and was increased significantly after co-transfection of PDK4-WT (wild type) and miR-148 inhibitor (Figure 5e). However, luciferase activity was not affected by both miR-148 agomir and miR-148 inhibitor in PDK4 – mutate (MUT) group. At the same time, the RNA pull down assay showed that endogenous PDK4 was enriched specifically in miR-148 probe detection compared with control group, further indicating the targeting relationship between PDK4 and the miR-148 (figure 5f). Furthermore, siRNA targeting PDK4 in HEK-293 T cells was constructed. RT-qPCR showed that the expression of PDK4 was significantly suppressed after the transfection of siPDK4, indicating that the suppression system was successfully constructed (Figure 5g). In addition, miR-148 agomir and/or siPDK4 were transfected into HEK-293 T cells, and western blot was used to detect the expression of PDK4. The results showed that inhibiting effect of miR-148 inhibitor on the expression of PDK4 was blocked in the presence of siPDK4 (Figure 5h). Moreover, expressions of miR-148 and PDK4 had negative correlation as shown in Figure 5i. Therefore, there exists a negative correlation between miR-148 and PDK4.

### Protective effect of down-regulated PDK4 on cardiac dysfunction

**Figure 6.**
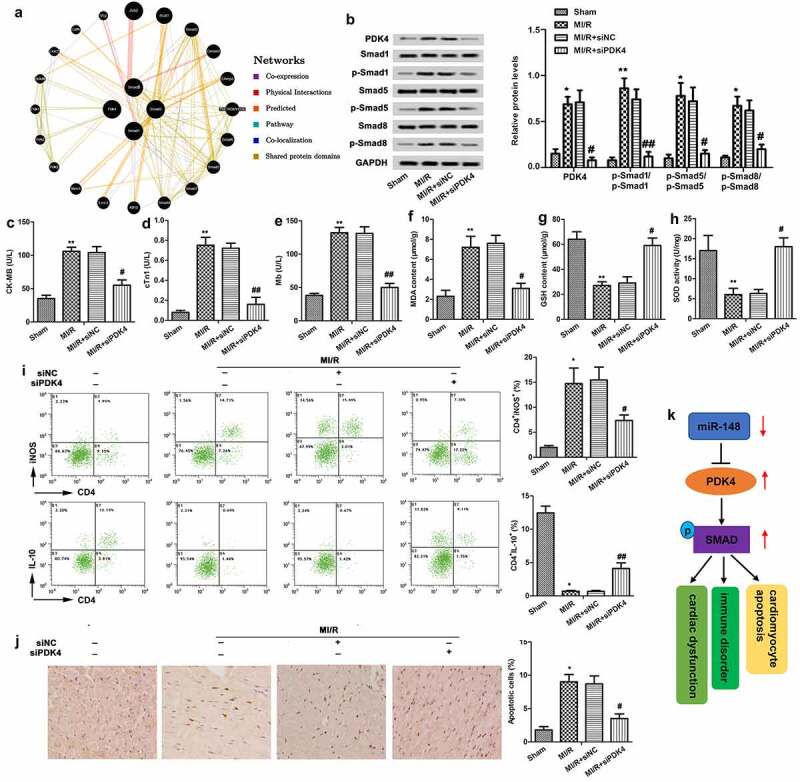
**Protective effect of down-regulated PDK4 on cardiac dysfunction**. Immature rats with MI/R injury were transfected with siNC or siPDK4 or left untreated as the model control. (a) The gene network diagram obtained from GeneMania showed the various genes within the SMAD pathway that are affected by PDK4. (b) Protein levels of SMAD pathway related p-smad1/smad1, p-smad5/smad5 and p-smad8/smad8 were detected through western blot. (c) CK-MB value. (d) cTn1 value. (e) Mb value. (f) The content of MDA. (g) The content of GSH. (h) The content of SOD. (i) CD4^+^iNOS^+^ and CD4^+^IL-10^+^ cells in peripheral blood were sorted by flow cytometry. (j) Cell apoptosis was measured by TUNEL staining. (k) The graphical abstract showed that miR-148 regulated the cardiac dysfunction, immune disorders and myocardial apoptosis by targeting PDK4 via the SMAD pathway. The data is shown as means ± SD, *P < 0.05, **P < 0.01 vs. control group, ^#^*P* < 0.05 vs. MI/R + Ad-PDK4 group, and ns means no significant difference

The individual effects of PDK4 on cardiac dysfunction were also explored. Firstly, a gene network diagram obtained from GeneMania showed various genes within the SMAD pathway which are affected by PDK4 (Figure 6a). Then, we found that the SMAD pathway was inactivated by the knockdown of PDK4 through suppressing the phosphorylation of smad1, smad5 and smad8 (Figure 6b). Further functional analysis showed that knockdown of PDK suppressed the levels of CK-MB (Figure 6c), cTn1 (Figure 6d) and Mb (Figure 6e) and CD4+ iNOS^+^ cells (Figure 6i) in peripheral blood, while elevated the level of CD4+ IL-10^+^ cells (Figure 6i), suggesting that silencedPDK4 exerted cardioprotective and immunomodulatory effects. In addition, elevated expression level of MDA and suppressed expression level of GSH and SOD were all reversed by silenced PDK4, demonstrating that silenced PDK4 showed anti-oxidation activity (figure 6f-h). Moreover, knockdown of PDK4 decreased cardiomyocyte apoptosis (Figure 6j). In addition, a graphical abstract showed that PDK4 was targeted by miR-148 and the SMAD pathway was activated by the induction of PDK4. The miR-148/PDK4/SMAD pathway relieved the MI/R injury through regulating the cardiac dysfunction, immune disorders and myocardial apoptosis (Figure 6k). The above experiments showed that knockdown of PDK4 attenuated cardiac dysfunction, immune disorder, and apoptosis and restored the anti-oxidation activity in immature rats with MI/R injury via the inactivation of SMAD pathway.

## DISCUSSION

Inadequate blood supply to the myocardium leads to damage in the structure and function of cardiomyocytes [25]. Clinically, reperfusion therapy is often employed to restore blood supply rapidly [26]. However, rapid supply of myocardial blood flow can exacerbate myocardial damage, leading to cardiomyocyte apoptosis, arrhythmias, and impaired cardiac function [27]. Up to now, miRNAs have been widely explored in various diseases. Previous study has also pointed out the neuroprotective effect of miR-22 in I/R injury [28]. However, association between miRNA and immume system in I/R injury has not been widely explored. In this study, cardiac dysfunction, immune dysfunction and myocardial cell apoptosis in immature rats were induced by MI/R treatment. Fortunately, abnormal cardiac function index, myocardial injury index and myocardial tissue were all recovered to the normal levels in the presence of overexpressed miR-148 in normal immature rats.

MiR-148 not only plays a critical role in differentiation and immune response in immune cell, but also is involved in the occurrence and development of many tumor and non-tumor diseases [29]. Many studies have identified that miR-148 family act as oncogenes or tumor suppressor genes [30]. In addition, accumulated evidence indicates that miR-148 also plays an important role in some non-tumor diseases, such as IgA nephropathy, type 2 diabetes (T2D) and liver injury, etc. In IgA nephropathy patients, miR-148b induced o-glycosylation abnormalities by targeting C1GALT1 [31]. Studies by Lopez et al have shown that miR-148a is significantly elevated in the blood circulation in patients with T2D [32]. In addition, the expression of miR-148a in liver tissues was significantly decreased with the extension of the duration of hot ischemia after transplantation [33]. Chavali et al. found that the low expression of miR-148 was associated with heart failure in Insulin2 mutant Akita model mice [34]. In addition, miR-148 was considered as the top regulatory factor of cardiac regeneration and played an important role in the regulation of cardiomyocyte proliferation [35]. In this study, we found that lowly expressed miR-148 in immature MI/R rats induced cardiac dysfunction, immune dysfunction and cardiomyocyte apoptosis. These abnormalities were mitigated by forcing overexpression of miR-148. Further molecular analysis showed that miR-148 exerted a protective effect on immature rats after MI/R injury by inhibiting the expression of PDK4. In summary, our results emphasize the protective effects of miR-148 on MI/R injury in immature rats and its potential molecular mechanism, which provides a theoretical basis for clinical diagnosis and treatment of MI/R injury in immature rats.

CD4 + T lymphocytes are human effectors involved in inflammatory immune-mediated diseases [36]. Interleukin-10 (IL-10) is a typical anti-inflammatory cytokine which promotes immune response [37]. Nitric oxide (NO) is an important pro-inflammatory medium, and inducible nitric oxide synthase (iNOS) is one of the three enzymes for the synthesis of NO [38]. Studies have shown that iNOS in T cells and macrophages regulates the differentiation and function of immune cells [39]. MiR-148A is essential for the normal regulation and function of T and B lymphocytes [40]. MiR-148a promotes the survival of repeatedly activated Th1 lymphocytes by suppressing the gene Bcl2L11 and encoding pro-apoptotic protein Bim [41], thus participating in immune regulation. Overexpression of miR-148 inhibits the production of lipopolysaccharide-induced inflammatory cytokines, such as IL-6 and IL-1β, thereby avoiding excessive inflammation [42]. Consistent with the above studies, this study found that miR-148 participated in immune regulation by reducing the level of CD4+ iNOS+ cells and the production of iNOS. Besides, increased number of CD4+ IL-10+ cells and the elevated levels of IL-10 in the peripheral blood of immature rats with MI/R injury further reveal a new mechanism through which miR-148 regulates the immune dysfunction in immature rats with MI/R injury.

Our study confirmed that PDK4 was a target gene of miR-148, and the expression of them two was negatively correlated. Pyruvate dehydrogenase kinase (PDK) regulates mammalian pyruvate dehydrogenase complex (PDC) through phosphorylation (activation) and dephosphorylation (inactivation). PDK4 is one of the four PDK proteins expressed in most tissues and organs [43]. DK4 plays an important role in inhibiting glucose oxidation by phosphorylating PDC [44]. It has also been reported that progesterone can induce the expression of PDK4 in cardiomyocytes and increased level of PDK4 in the third trimester of pregnancy further leads to cardiac hypertrophy during pregnancy [45]. Extracorporeal cardiac perfusion showed an increase in PDK4 levels in mouse hearts treated with angiotensin II (Ang II), while the absence of PDK4 prevented angiotensin converting enzyme II–induced diastolic dysfunction [46]. In this study, PDK4, a downstream factor of miR-148, was found to be involved in the cardio-protection of immature rats with MI/R injury. It was obvious that the up-regulation of PDK4 aggravated cardiac dysfunction, immune dysfunction and cardiomyocyte apoptosis in immature rats with MI/R injury. The expression of PDK4 was inhibited by overexpressed miR-148, which further reduced the MI/R injury of PDK4 in immature rats. Our study demonstrated that PDK4 could be used as a potential target for clinical diagnosis and treatment of pediatric heart disease.

It has been confirmed that TGF-β1 pathway plays an important role in the process of remodeling after myocardial infarction. Smads protein is an important downstream regulator of TGF-β1 signaling pathway. TGF-β1 is phosphorylated and activated after binding with its receptor Smads protein, which can play the role of transcription factors and regulate the gene expression of related cytokines [16,21]. In our study, expressions of phosphorylated smad1, smad5 and smad8 were elevated in MI/R group. Then, the elevated expression of phosphorylated smad1, smad5 and smad8 was effectively suppressed by silenced PDK4. Thus, the protective effects of siPDK4 were accompanied by the inactivation of Smads proteins.

## CONCLUSIONS

Taken together, the results of this study suggest that overexpressed miR-148 can reduce cardiac dysfunction, immune disorder and cardiomyocyte apoptosis in immature MI/R rats by targeting PDK4 and inhibiting its expression. Besides, the protective effects of silenced PDK4 may be accompanied by inactivation of SMAD pathway. This study provides some ideas and data support for the treatment of ischemic heart disease, especially in children.

## Data Availability

The datasets used and/or analyzed during the current study are available from the corresponding author on reasonable request.

## Highlights

1. MiR-148 agomir alleviates cardiac dysfunction in MI/R injury

2. MiR-148 agomir alleviates immune disorder and cardiomyocyte apoptosis caused by MI/R.

3. Protective effect of MiR-148 via PDK4 down-regulation.

## References

[1] McCusker CG, Doherty NN, Molloy B, et al. A randomized controlled trial of interventions to promote adjustment in children with congenital heart disease entering school and their families. J Pediatr Psychol. 2012;37(10):1089–1103. 22976507 10.1093/jpepsy/jss092

[2] van Rijen EHM, Utens EMWJ. Roos-Hesselink JW et al. Psychosocial functioning of the adult with congenital heart disease: a 20-33 years follow-up. Eur Heart J. 2003;24(7):673–683. 12657226 10.1016/s0195-668x(02)00749-2

[3] Spijkerboer AW, Utens EMWJ, Bogers AJJC, et al. A historical comparison of long-term behavioral and emotional outcomes in children and adolescents after invasive treatment for congenital heart disease. J Pediatr Surg. 2008;43(3):534–539. 18358296 10.1016/j.jpedsurg.2007.10.037

[4] Rezende PC, Ribas FF, Serrano CV, et al. Clinical significance of chronic myocardial ischemia in coronary artery disease patients. J Thorac Dis. 2019;11(3):1005–1015. 31019790 10.21037/jtd.2019.02.85 PMC6462715

[5] Mokhtari-Zaer A, Marefati N, Atkin SL, et al. The protective role of curcumin in myocardial ischemia-reperfusion injury. J Cell Physiol. 2018;234(1):214–222. 29968913 10.1002/jcp.26848

[6] Lu TX, Rothenberg ME. MicroRNA. J Allergy Clin Immunol. 2018;141(4):1202–1207. 29074454 10.1016/j.jaci.2017.08.034 PMC5889965

[7] Vp Y, Lepilina A, Smith A, et al. Regulation of zebrafish heart regeneration by miR-133. Dev Biol. 2012;365(2):319–327. Beauchemin M, Smith A, Yin VP. Dynamic microRNA-101a and Fosab expression controls zebrafish heart regeneration *Development*. 2015;142:4026-4037. 22374218 10.1016/j.ydbio.2012.02.018 PMC3342384

[8] Crippa S, Nemir M, Ounzain S, et al. Comparative transcriptome profiling of the injured zebrafish and mouse hearts identifies miRNA-dependent repair pathways. Cardiovasc Res. 2016;110(1):73–84. 26857418 10.1093/cvr/cvw031 PMC4798047

[9] Jiang C-K, Gong F. [Regulation and mechanism of miR-148a on cardiomyocyte differentiation induced by 5-aza in mesenchymal stem cells]. Zhongguo Ying Yong Sheng Li Xue Za Zhi. 2017;33:514–518. 29931900 10.12047/j.cjap.5601.2017.122

[10] Zhang J, Ying Z-Z, Tang Z-L, et al. MicroRNA-148a promotes myogenic differentiation by targeting the ROCK1 gene. J Biol Chem. 2012;287(25):21093–21101. 22547064 10.1074/jbc.M111.330381 PMC3375532

[11] Klett H, Jürgensen L, Most P, et al. Delineating the dynamic transcriptome response of mRNA and microRNA during zebrafish heart regeneration. Biomolecules. 2018;9(1):11. 10.3390/biom9010011 PMC6359357 30597924

[12] Toldo S, Mauro AG, Cutter Z, et al. Inflammasome, pyroptosis, and cytokines in myocardial ischemia-reperfusion injury. Am J Physiol Heart Circ Physiol. 2018;315(6):H1553–h1568. 30168729 10.1152/ajpheart.00158.2018 PMC6336966

[13] Giri BR, Cheng G. Host miR-148 regulates a macrophage-mediated immune response during Schistosoma japonicum infection. Int J Parasitol. 2019;49(13–14):993–997. 31726056 10.1016/j.ijpara.2019.08.002

[14] Sheeran FL, Angerosa J, Liaw NY, et al. Adaptations in protein expression and regulated activity of pyruvate dehydrogenase multienzyme complex in human systolic heart failure. Oxid Med Cell Longev. 2019;2019:4532592. 10.1155/2019/4532592 PMC6383428 30881593

[15] Li T, Xu J, Qin X, et al. Glucose oxidation positively regulates glucose uptake and improves cardiac function recovery after myocardial reperfusion. American Journal of Physiology. Endocrinology and Metabolism. 2017;313(5):E577–e585. 28325730 10.1152/ajpendo.00014.2017

[16] Xue R, Lei S, Xia ZY, et al. Selective inhibition of PTEN preserves ischaemic post-conditioning cardioprotection in STZ-induced Type 1 diabetic rats: role of the PI3K/Akt and JAK2/STAT3 pathways. Clin Sci (Lond). 2016;130(5):377–392. 26666444 10.1042/CS20150496

[17] Qiu Z, Lei S. NLRP3 inflammasome activation-mediated pyroptosis aggravates myocardial ischemia/reperfusion injury in diabetic rats. Oxid Med Cell Longev. 2017;2017:9743280. 10.1155/2017/9743280 PMC5618779 29062465

[18] Yu T, Zhao C. Exosomes secreted from miRNA-29b-modified mesenchymal stem cells repaired spinal cord injury in rats. Braz J Med Biol Res. 2019;52:e8735. 10.1590/1414-431X20198735 PMC6903804 31826179

[19] Yu SY, Dong B, Fang ZF, et al. Knockdown of lncRNA AK139328 alleviates myocardial ischaemia/reperfusion injury in diabetic mice via modulating miR-204-3p and inhibiting autophagy. J Cell Mol Med. 2018;22:4886–4898. 10.1111/jcmm.13754 PMC6156366 30047214

[20] Wang H, Huo X, Yang XR, et al. STAT3-mediated upregulation of lncRNA HOXD-AS1 as a ceRNA facilitates liver cancer metastasis by regulating SOX4. Mol Cancer. 2017;16(1):136. 28810927 10.1186/s12943-017-0680-1 PMC5558651

[21] Sadeghi S, Hojati Z, Tabatabaeian H. Cooverexpression of EpCAM and c-myc genes in malignant breast tumours. J Genet. 2017;96(1):109–118. 28360395 10.1007/s12041-017-0748-0

[22] Zhang B, Shetti D, Fan C, et al. miR-29b-3p promotes progression of MDA-MB-231 triple-negative breast cancer cells through downregulating TRAF3. Biol Res. 2019;52:38. 10.1186/s40659-019-0245-4 PMC6659300 31349873

[23] An X, Ma H, Liu Y, et al. Effects of miR-101-3p on goat granulosa cells in vitro and ovarian development in vivo via STC1. J Anim Sci Biotechnol. 2020;11:102. 10.1186/s40104-020-00506-6 PMC7557009 33072314

[24] Chen D, Zheng X, Kang D, et al. Apoptosis and expression of the Bcl-2 family of proteins and P53 in human pancreatic ductal adenocarcinoma. Med Princ Pract. 2012;21(1):68–73. 22024503 10.1159/000332423

[25] Marín-Juez R, El-Sammak H, Helker CSM, et al. Coronary revascularization during heart regeneration is regulated by epicardial and endocardial cues and forms a scaffold for cardiomyocyte repopulation. Dev Cell. 2019;51(4):503–515.e4. 10.1016/j.devcel.2019.10.019 PMC6982407 31743664

[26] Bhaskar S, Stanwell P, Cordato D, et al. Reperfusion therapy in acute ischemic stroke: dawn of a new era? BMC Neurol. 2018;18:8. 29338750 10.1186/s12883-017-1007-y PMC5771207

[27] Jahan R, Saver JL, Schwamm LH, et al. Association between time to treatment with endovascular reperfusion therapy and outcomes in patients with acute ischemic stroke treated in clinical practice. JAMA. 2019;322(3):252–263. 31310296 10.1001/jama.2019.8286 PMC6635908

[28] Jiao H, Chen R, Jiang Z, et al. miR-22 protect PC12 from ischemia/reperfusion-induced injury by targeting p53 upregulated modulator of apoptosis (PUMA). Bioengineered. 2020;11(1):209–218. 32065044 10.1080/21655979.2020.1729321 PMC7039629

[29] Babu KR, Muckenthaler MU. miR-148a regulates expression of the transferrin receptor 1 in hepatocellular carcinoma. Sci Rep. 2019;9(1):1518. 30728365 10.1038/s41598-018-35947-7 PMC6365501

[30] Chen Y, Song Y-X, Wang Z-N. The microRNA-148/152 family: multi-faceted players. Mol Cancer. 2013;12(43):43. 10.1186/1476-4598-12-43 PMC3671164 23683438

[31] Serino G, Sallustio F, Cox SN, et al. Abnormal miR-148b expression promotes aberrant glycosylation of IgA1 in IgA nephropathy. J Am Soc Nephrol. 2012;23:814–824. 22362909 10.1681/ASN.2011060567 PMC3338289

[32] Nunez Lopez YO, Garufi G, Seyhan AA. Altered levels of circulating cytokines and microRNAs in lean and obese individuals with prediabetes and type 2 diabetes. Mol Biosyst. 2016;13(1):106–121. 27869909 10.1039/c6mb00596a

[33] Farid WRR, Pan Q, van der Meer AJP, et al. Hepatocyte-derived microRNAs as serum biomarkers of hepatic injury and rejection after liver transplantation. Liver Transpl. 2012;18(3):290–297. 21932376 10.1002/lt.22438

[34] Chavali V, Tyagi SC, Mishra PK. Differential expression of dicer, miRNAs, and inflammatory markers in diabetic Ins2± Akita hearts. Cell Biochem Biophys. 2014;68(1):25–35. 23797610 10.1007/s12013-013-9679-4 PMC4085798

[35] Klett H, Jürgensen L. Delineating the dynamic transcriptome response of mRNA and microRNA during zebrafish heart regeneration. Biomolecules. 2018;9 (1):11. 10.3390/biom9010011 PMC6359357 30597924

[36] Cosmi L, Maggi L, Santarlasci V, et al. T helper cells plasticity in inflammation. Cytometry A. 2014;85(1):36–42. 24009159 10.1002/cyto.a.22348

[37] Bedke T, Muscate F, Soukou S, et al. Title: IL-10-producing T cells and their dual functions. Semin Immunol. 2019;44:101335. 31734129 10.1016/j.smim.2019.101335

[38] Xue Q, Yan Y, Zhang R, et al. Regulation of iNOS on immune cells and its role in diseases. Int J Mol Sci. 2018;19(12):3805. 10.3390/ijms19123805 PMC6320759 30501075

[39] Mishra BB, Rathinam VAK, Martens GW, et al. Nitric oxide controls the immunopathology of tuberculosis by inhibiting NLRP3 inflammasome-dependent processing of IL-1β. Nat Immunol. 2013;14(1):52–60. 23160153 10.1038/ni.2474 PMC3721324

[40] Friedrich M, Pracht K, Mashreghi M-F, et al. The role of the miR-148/-152 family in physiology and disease. Eur J Immunol. 2017;47(12):2026–2038. 28880997 10.1002/eji.201747132

[41] Haftmann C, Stittrich A-B, Zimmermann J, et al. miR-148a is upregulated by twist1 and T-bet and promotes Th1-cell survival by regulating the proapoptotic gene bim. Eur J Immunol. 2015;45(4):1192–1205. Haftmann C, Riedel R, Porstner Met al. Direct uptake of Antagomirs and efficient knockdown of miRNA in primary B and T lymphocytes *J Immunol Methods*. 2015;426:128-133. 25486906 10.1002/eji.201444633 PMC4406154

[42] Chu Q, Gao Y, Bi D, et al. MicroRNA-148 as a negative regulator of the common TLR adaptor mediates inflammatory response in teleost fish. Sci Rep. 2017;7(1):4124. 28646187 10.1038/s41598-017-04354-9 PMC5482802

[43] Mc S, MJ H. Therapeutic potential of the mammalian pyruvate dehydrogenase kinases in the prevention of hyperglycaemia. Curr Drug Targets Immune Endocr Metabol Disord. 2002;2(2):151–165. 12476789

[44] Ushikai M, Horiuchi M, Kobayashi K, et al. Induction of PDK4 in the heart muscle of JVS mice, an animal model of systemic carnitine deficiency, does not appear to reduce glucose utilization by the heart. Mol Genet Metab. 2011;102(3):349–355. 21190881 10.1016/j.ymgme.2010.11.167

[45] Liu LX, Rowe GC, Yang S, et al. PDK4 inhibits cardiac pyruvate oxidation in late pregnancy. Circ Res. 2017;121(12):1370–1378. 28928113 10.1161/CIRCRESAHA.117.311456 PMC5722682

[46] Mori J, Alrob OA, Wagg CS, et al. ANG II causes insulin resistance and induces cardiac metabolic switch and inefficiency: a critical role of PDK4. Am J Physiol Heart Circ Physiol. 2013;304(8):H1103–H1113. 23396452 10.1152/ajpheart.00636.2012

